# Fitting Geometric Shapes to Fuzzy Point Cloud Data

**DOI:** 10.3390/jimaging11010007

**Published:** 2025-01-03

**Authors:** Vincent B. Verhoeven, Pasi Raumonen, Markku Åkerblom

**Affiliations:** Faculty of Information Technology and Communication Sciences, Mathematics Research Centre, Tampere University, Korkeakoulunkatu 1, 33720 Tampere, Finland; markku.akerblom@tuni.fi

**Keywords:** uncertainty quantification, geometry reconstruction, laser scanning, point cloud

## Abstract

This article describes procedures and thoughts regarding the reconstruction of geometry-given data and its uncertainty. The data are considered as a continuous fuzzy point cloud, instead of a discrete point cloud. Shape fitting is commonly performed by minimizing the discrete Euclidean distance; however, we propose the novel approach of using the expected Mahalanobis distance. The primary benefit is that it takes both the different magnitude and orientation of uncertainty for each data point into account. We illustrate the approach with laser scanning data of a cylinder and compare its performance with that of the conventional least squares method with and without random sample consensus (RANSAC). Our proposed method fits the geometry more accurately, albeit generally with greater uncertainty, and shows promise for geometry reconstruction with laser-scanned data.

## 1. Introduction

In a typical shape-fitting problem with data covering the object’s surface (a so-called point cloud), the distance between the points and the shape is used as the metric to quantify how well a given shape fits the data. For example, in the least squares approach the sum of the squared distances is used as the objective function to be minimized in order to find the optimal shape parameters for the fitting problem. However, this approach does not consider the inherent uncertainties in the measured point cloud data explicitly.

There are many sources of point location uncertainty, and moreover, the uncertainty can vary a lot between the data points. For example, in light detection and ranging (LiDAR) instruments, such as fixed position terrestrial laser scanning (TLS) instruments, the point location uncertainty is affected by the range and resulting beam size and the incidence angle between the laser beam and the measured object’s surface.

In this paper, we present a generalization of the shape-fitting problem with point cloud data which considers the assumed/known uncertainty of each data point. In this generalization, we model the uncertainty of each data point explicitly with three-dimensional (3D) distributions such as Gaussians, which may be of varying magnitude and orientation to account for the differences in uncertainty between the data points. Thus, our data set for the fitting problem is not a 3D point cloud but a so-called *fuzzy point cloud*; a cloud of 3D distributions, and each of these distributions can be different in size and shape. This more accurately describes the underlying data and should therefore enable a more accurate fit of the geometry and the ability to determine its uncertainty. The outlined approach comes from the field of uncertainty quantification and is an improvement over other Bayesian methods that assume homoscedasticity [1], assume the distance to the geometry to follow a Gaussian distribution [1,2], or assume a distribution of the geometry parameters [3]. Additionally, the method requires no uniform sampling or full coverage of the geometry.

The distance between the points and the shape to be reconstructed is replaced with the expected distance between the distributions and the shape. The distance metric does not need to be the Euclidean distance and is instead substituted by the Mahalanobis distance to incorporate anisotropic uncertainty, i.e., [4]. To the best knowledge of the authors, the application of the Mahalanobis distance for geometry fitting is rare. Our approach deviates from an existing study in the approach to reduce bias and by evaluating the entire geometry for every distribution rather than the Mahalanobis nearest point [5]. Finally, the objective function for the shape-fitting problem is the average of the expected distances over the fuzzy point cloud.

Geometry reconstruction of laser-scanned objects has been applied in both the built and natural environment in a large body of research [6,7,8,9]. As such, we demonstrate the method in detail by fitting a cylinder to simulated LiDAR data. The choice for this shape is motivated by its commonality in both the natural and built environment, for instance, to approximate part of a tree or a pipe [10,11].

We start this paper by describing the fuzzy data in Section 2, followed by our geometry fitting approach in Section 3. The aforementioned approach is illustrated on cylinder fitting with laser scanning data in Section 4, followed by a discussion in Section 5, and finally, the conclusion in Section 6.

## 2. Fuzzy Data

### 2.1. Fuzzy Point Clouds

**Figure 1:**
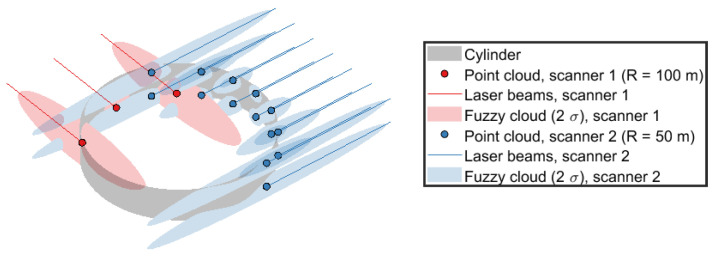
Example fuzzy point cloud generated for a cylinder slice scanned by two laser scanners at a range of 50 m and 100 m, respectively, 90 degrees apart.

The data consist of a fuzzy point cloud FC which is a generalization of a point cloud: Instead of having a finite set of points *X* in $R^{m}$ (typically $R^{3}$), we have a finite set of *n* distributions $N_{i}$ defined over $R^{m}$, i.e. $FC=\{N_{i}\;|\;i=1,2,\ldots ,n\}$. Notice that each distribution can be different in shape and variance. Some can be finitely supported and others have infinite support, but the support is *m*-dimensional. To model laser scanning in the real world, the distributions consist of trivariate normal distributions given by $N_{i}=N_{i}\left({\hat{\mu }}_{i},\sum_{i}\right)$, where ${\hat{\mu }}_{i}=\left[x_{i},y_{i},z_{i}\right]\in X\subset R^{3}$ is the *i*th measured point and $\sum_{i}$ its covariance matrix, modeling the uncertainty of the measured location. Thus, the advantage and difference in using fuzzy point clouds as opposed to point clouds is that we can rigorously consider the uncertainty of each point, which locally can vary significantly depending on the geometrical shape. An example fuzzy point cloud is shown in Figure 1 for a cylinder section measured by two laser scanners.

### 2.2. Determination of Fuzziness

One important and immediate question regarding fuzzy point clouds is the ability to define the distributions for a given problem. For example, if the data come from laser scanners, then the uncertainty of the measurement not only depends on the instrument’s specifications but also on the distance (range) to the object. When the range of the object is much greater than its dimensions, it may be assumed independent of the object’s exact geometry and considered a constant. More importantly, however, is the incidence angle between the laser beam and the measured object’s surface, which is often not known, at least not accurately. We can perhaps estimate quite accurately the minimum uncertainty for each point, e.g., in the case of laser scanning by assuming zero incidence angle, or if the object being measured is large enough so that small changes in the laser beam locations lead only to insignificant changes in the incidence angle so that the angle can be estimated well. Otherwise, we could have a situation in which uncertainty is very sensitive to the shape whose determination is the objective of the fitting problem. This circular relationship is discussed in more detail in the next section.

## 3. Geometry Fitting

This section describes the approach used to fit the optimal geometry to a fuzzy point cloud. It is written in a general way, such that it is independent of the geometric shape being fitted and is not specific to laser scanning data.

### 3.1. Objective Function

There are many possibilities for formulating meaningful objective functions for our shape-fitting problem. First, the basic idea is to minimize some distance between the data and the shape by varying the shape parameters. We have multiple choices for the distance $d:R^{m}\times R^{m}\to R$, such as the often used *Euclidean distance*, but also others, such as the *Mahalanobis distance*.

Because our data consist of continuous distributions and not discrete points, we need to define how the distance is defined between a distribution and a shape. With points, this is simply the minimum distance from the point to the shape. With distributions, we can similarly define the minimum distance from any point within the distribution to the shape and then integrate these distances over the support of the distribution to produce a single distance. A natural choice is the expected distance, where this point-wise minimum distance is weighted by the distribution’s probability density value.

Let *g* denote the set of geometry parameters describing the shape $G\subset R^{m}$, where a point lying on its surface is denoted by $\hat{p}\in G$. The minimum distance from a point $\hat{x}\in R^{m}$ to the shape G is then denoted by $d_{G}\left(\hat{x}\right)=\min_{\hat{p}\in G}d\left(\hat{p}\;|\;\hat{x}\right)$. Then, the expected distance from a distribution N with probability density $f(\hat{x})$ to the shape *G* is given by

(1) $$E\left[d_{G}\left(N\right)\right]=\int_{\mathrm{supp}(N)}f\left(\hat{x}\right)d_{G}\left(\hat{x}\right)d\hat{x}.$$

A natural alternative to the expected distance $E[d_{G}\left(N_{i}\right)]$ with distributions $N_{i}$ would be the maximum likelihood

(2) $$L\left(G|N_{i}\right)=\max_{\hat{p}\in G}N_{i}\left(\hat{p}\right).$$

The fit is then quantified by how likely each geometry is for a given distribution. The objective function would be the mean of the likelihoods over the data. However, we use the expected distance instead of the maximum likelihood despite it perhaps being computationally easier to determine, because the objective function based on the likelihood suffers the major problem that the likelihood values can easily become negligible, and thus, their contribution to the objective function and indeed its gradient become negligible. This would result in said distribution being ignored by the optimizer, hence our choice to use the expected distance. Similarly, the log-likelihood suffers from extreme gradients for low likelihood values.

The objective function O(shape|data)=O(G|FC) is the mean of the expected distances over the fuzzy point cloud FC. The optimal geometry is thus the one that minimizes the following sum:

(3) $$\arg\min_{G}O\left(G\;|\;FC\right)=\arg\min_{G}\frac{1}{n}\sum_{i=1}^{n}E\left[d_{G}\left(N_{i}\right)\right].$$

For non-trivial problems, this minimization problem is highly complex and thus solved iteratively, starting from an initial geometry estimate $G_{0}$, from which the final geometry estimate *G* follows. To make the objective function values comparable irrespective of the number of points or uncertainty magnitude, the objective function is divided by the value given by the initial estimate. The geometry fitting problem can now be formulated as the minimization problem

(4) $$\arg\min_{G}O\left(G\;|\;FC\right)=\arg\min_{G}\frac{\sum_{i=1}^{n}E\left[d_{G}\left(N_{i}\right)\right]}{\sum_{i=1}^{n}E\left[d_{G_{0}}\left(N_{i}\right)\right]}.$$

### 3.2. Mahalanobis Distance

We alluded already that one possible and often used distance metric for shape fitting is the Euclidean distance; however, it has some potential problems: First, it is scale-dependent. More natural would be to have the distance metric depend on the magnitude of the uncertainty, i.e., how far a point is from the shape in terms of standard deviations of the distributions, instead of it depending on arbitrary units such as meters or inches. Second, the Euclidean distance is symmetric or isotropic, meaning it treats every direction equally. We can, however, generally not assume the uncertainty to be isotropic due to anisotropy in measurements, and thus, it makes sense for the distance function to also include this possible anisotropy. A more suitable distance function is the Mahalanobis distance. Given two points $\hat{x},\hat{y}\in R^{m}$, the Mahalanobis distance $M_{i}$ for the distribution $N_{i}$ is defined as

(5) $$M_{i}\left(\hat{x},\hat{y}\right)=\sqrt{{\left(\hat{x}-\hat{y}\right)}^{T}P_{i}\left(\hat{x}-\hat{y}\right)},$$

where $P_{i}$ is the m×m precision matrix (i.e., the inverse of the positive-definite covariance matrix $\sum_{i}$) of distribution $N_{i}$.

The Mahalanobis distance is unitless, scale-invariant, and considers the directional dependence (anisotropy) of the distribution’s covariance structure. We note that the definition is a unitless equivalent of the Euclidean distance when the distribution’s covariance matrix equals the identity matrix.

### 3.3. Uncertainty Independent of Geometry

The expected values ${\hat{\mu }}_{i}\in X$ are the measured point locations, and thus our input data for shape fitting; however, the issue is raised that the covariance of the distributions depends not only on the given point locations but also on the unknown geometry, i.e., $\sum_{i}=f\left({\hat{\mu }}_{i},G\right)$. Contrary to the Euclidean distance, the Mahalanobis distance depends on the covariance, so we have a cyclical problem G=h(X,G), where the function *h* denotes the minimization of the objective function.

**Figure 2:**
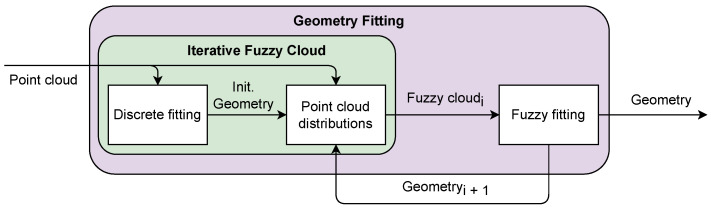
General fuzzy geometry fitting flowchart. Index i denotes fuzzy point cloud iteration.

An intuitive way to break this cycle is to make the distributions depend on a previous estimate of the geometry, especially as a minimization of the objective function is an iterative process. However, making the distributions dependent on previous iterations leads to several issues in minimizing the objective function that are outside of the scope of this paper. Instead, we first provide an initial estimate of the geometry using the discrete point cloud, i.e., through least squares, which we use to create the fuzzy point cloud used for fitting. As the distributions may be sensitive to the initial geometry estimate, the optimized geometry is used iteratively to update the fuzzy point cloud until the geometry has converged. A general flowchart of the full approach for fuzzy geometry fitting is presented in Figure 2.

## 4. Cylinder Fitting with Laser Scanning Data as an Example

To illustrate the described approach, it is applied to the case of cylinder fitting to laser scanning point cloud data. The circular cross-sectional shape results in a strongly variable incidence angle and thus varying local uncertainty between the points. We further note that most geometry in the built and natural environment can be approximated to be locally of constant cross-section making this an acceptable simplification.

The cylinder is parameterized by its radius *r*, its azimuth and elevation angles, and its center ${\hat{c}}_{0}$. We note that for a given azimuth and elevation angle (i.e., cylinder axis), the cylinder center may be projected onto the cross-sectional reference plane $Q⊥\vec{v}$ s.t. its dimensionality is now two, i.e., ${\hat{c}}_{Q}={\mathrm{proj}}_{Q}\left({\hat{c}}_{0}\right)\in R^{2}$. For the intent of this paper, the cylinder’s length *l* is ignored. The approach to reduce the problem’s dimensionality is applicable to any shape of constant cross-section and is given in the Appendix A.

### 4.1. Data

Using the Gaussian approximation for the power within a laser beam, the standard deviation in the radial direction $\sigma_{radial}$ given by Equation (6) is a quarter of the beam diameter $d_{B}$, as it is commonly defined as covering four standard deviations [8]. It has an initial exit diameter $d_{0}$ and increases according to the beam divergence half-angle λ. As the range *R* between the measured point $\hat{\mu }$ and scanner $\hat{s}$ can safely be assumed to be much greater than its Rayleigh length, the increase in diameter is approximately linear.

(6) $$\begin{array}{c} \sigma_{radial}\left(\hat{\mu }\;|\;\hat{s}\right)=\frac{d_{B}}{4}=\frac{d_{0}+2R\tan\left(\lambda \right)}{4}=\\ \frac{d_{0}}{4}+\frac{1}{2}\left|\right|\hat{\mu }-\hat{s}\left|\right|\tan\left(\lambda \right). \end{array}$$

The standard deviation in the propagation direction $\sigma_{prop}$ can be seen as a ‘smearing out’ of the radial uncertainty due to the incidence angle α between the beam and the surface. It follows from the dot product between the vectors from $\hat{\mu }$ to its projection onto the cylinder axis $\hat{p}$ and the scanner location $\hat{s}$, respectively.

(7) $$\alpha \left(\hat{\mu }\;|\;\hat{s},\hat{c},\vec{v}\right)=\arccos\left(\frac{\left|\langle \hat{\mu }-\hat{p},\hat{\mu }-\hat{s}\rangle \right|}{R||\hat{\mu }-\hat{p}\left|\right|}\right)$$

Additionally, the propagation uncertainty $\sigma_{prop}$ includes $\sigma_{0}$ as the base-level range uncertainty of the device:

(8) $$\sigma_{prop}\left(\hat{\mu }\;|\;\hat{s},\hat{c},\vec{v}\right)=\sigma_{0}+\sigma_{radial}\left(\hat{\mu }\;|\;\hat{s}\right)\tan\left(\alpha \right).$$

This ensures that $\sigma_{prop}>0$, and thus that the Mahalanobis distance is finite.

Simulated data are used to evaluate the method. Two hundred random point clouds are sampled from an initial fuzzy point cloud created using the true geometry, which enables us to determine average and standard deviations for the geometry parameters. As an example, a section of a cylinder’s fuzzy point cloud from which these point clouds may be sampled is shown in Figure 1.

### 4.2. Objective

As already mentioned in Section 3.2, the (squared) Mahalanobis distance equals the unitless (squared) Euclidean distance when the distribution’s covariance matrix equals the identity matrix. For this reason, as well as the simplification of future calculations, the Gaussian distribution *N* is transformed by *T* to a standard normal *S*, i.e., T:N↦S. The Mahalanobis distance between point $\hat{x}$ and the cylinder’s two-dimensional representation *C* is then equal to the Euclidean distance *D* in the transformed problem, i.e., $M\left(\hat{x},\hat{p}\in C\right)=D\left(T\left(\hat{x}\right),T\left(\hat{p}\right)\in T\left(C\right)\right)=D\left(\hat{a},\hat{e}\in E\right)$. The Euclidean distance from any point $\hat{a}$ to the resulting ellipse follows from the method described in [12], where the nearest point on the ellipse may be found using Ferrari’s method [13]. This method, however, prohibits an analytical expression for the expected distance, and additionally, it is inherently biased as will be discussed in Section 5.

**Figure 3:**
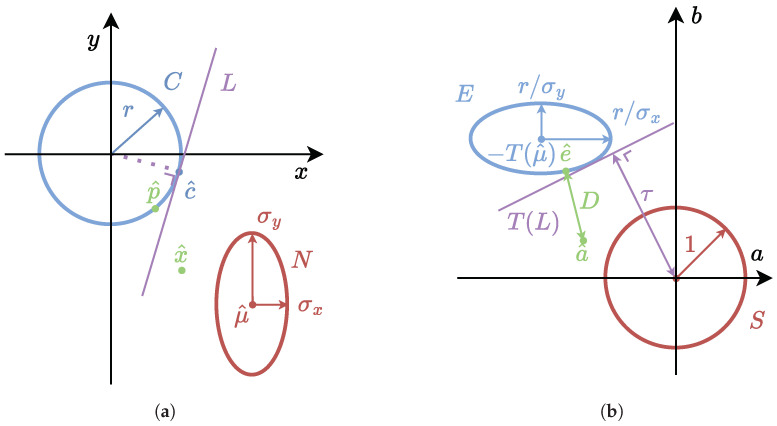
Illustration of the procedure to determine the expected Mahalanobis distance between a Gaussian distribution (**red**) and a tangent line (**purple**) placed on the circle (**blue**) before (**a**) and after transformation to the standard normal (**b**). The Gaussian distribution *N* is defined by the expected value $\hat{\mu }$ and the standard deviations $\sigma_{x}$ and $\sigma_{y}$ in the *x*- and *y*-directions, respectively.

The circle *C* is therefore instead approximated by its envelope of tangent lines *L* with their point $\hat{c}\in C$. After translating and scaling the axes such that the distribution is a standard normal, the distance τ between $\hat{\mu }$ and the line is calculated and the expected squared Mahalanobis distance of this line follows from Equation (9), where we note that the subscripts denote the relevant dimension. The approach is illustrated in Figure 3, where without loss of generality axes x,y are aligned with the projected Gaussian’s axes with standard deviation $\sigma_{x}$ and $\sigma_{y}$, respectively. Said axes become a,b after transformation to the standard normal.

(9) $$\begin{array}{c} \tau =\frac{|c_{x}\left(c_{x}-\mu_{x}\right)+c_{y}\left(c_{y}-\mu_{y}\right)|}{\sqrt{\sigma_{x}^{2}c_{x}^{2}+\sigma_{y}^{2}c_{y}^{2}}},\\ E\left[M^{2}\left(L\;|\;\hat{\mu },\sum \right)\right]=\tau^{2}+1. \end{array}$$

**Figure 4:**
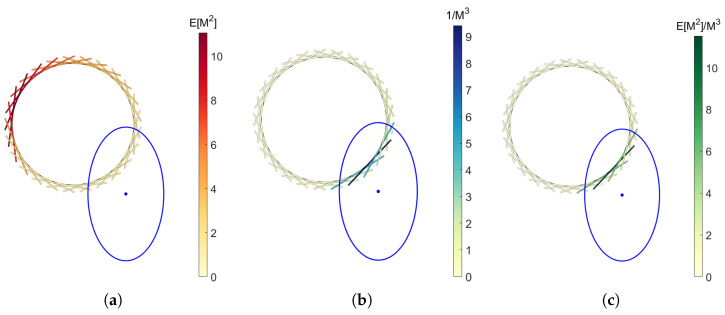
Illustration of the contributions of tangent lines in the envelope to (**a**) the expected Mahalanobis distance; (**b**) the inverse cubed Mahalanobis distance; and (**c**) the weighted expected Mahalanobis distance to the Gaussian distribution shown in blue (1σ). Note that the tangent lines are in reality infinite and all variables are dimensionless.

For the full envelope of *t* tangent lines, the expected squared Mahalanobis distance of each line is weighted by the distance of the point $\hat{c}$ to the distribution. The contribution of each tangent line to the objective function is illustrated in Figure 4. For clarity, the tangent lines are drawn as finite lines, and only 25 are shown versus the 1000 that were used in the actual computations. Care is taken that the Mahalanobis distance in the denominator is taken to a greater power than the expected Mahalanobis distance E[M], such that far-away tangent lines are weighed less even when $E\left[M\left(L_{i}\;|\;\hat{\mu },\sum \right)\right]\approx M\left({\hat{c}}_{i}\;|\;\hat{\mu },\sum \right)$.

(10) $$E\left[M^{2}\left(C\;|\;\hat{\mu },\sum \right)\right]=\frac{1}{t}\sum_{i=1}^{t}\frac{E[M^{2}\left(L_{i}\;|\;\hat{\mu },\sum \right)]}{M^{3}\left({\hat{c}}_{i}\;|\;\hat{\mu },\sum \right)}$$

### 4.3. Comparison with Least Squares

Geometry is often reconstructed from point cloud data using Euclidean distance least squares, i.e., for cylinder reconstruction [10,14]. Euclidean least squares (ELS) are assessed both with the full point cloud and using RANSAC, in which case it is abbreviated as RELS [15]. As the least squares fitting of a cylinder is a non-linear problem, an iterative Gauss–Newton approach is used to find the optimum. Similarly, an iterative interior-point algorithm is used to minimize the objective function using the envelope approach with the expected Mahalanobis (EM) distance described in this article. It is dependent on the initial geometry estimate, which came from ELS in which case the method is abbreviated as EM. If RANSAC was also used for the initial geometry estimate, the method is abbreviated to REM.

The approaches are compared for a cylinder with a radius and length of 5 and 25 cm, respectively, with the cylinder axis parallel to the *z*-axis. Scanner specifications correspond to the Faro Focus Plus series [16], however, at half the resolution (i.e., a quarter of the number of points). The scanner is located 50 and/or 100 m away from the cylinder. When two scanners are simulated, they are 90 degrees apart as seen from the cylinder.

The relative geometry fitting error (ν) and uncertainty (σ) are shown in Table 1. To increase the interpretability of the results, the values are divided by the true radius for the center and radius. As the vector is of unit length, no normalization is used there. For the purposes of this article, the location of the center along the cylinder axis (*z*-axis) and length are not relevant. Additionally, the error in the vector is fully described by the error of its *x* and *y* components. The dependent pairwise Student’s *t*-test is used to determine whether or not the difference between the methods is statistically significant and the resulting probability values *p* are given in Table 2.

The computational times of the 200 iterations are given in Table 3 and are meant only to give an idea of the relative computational time between the different methods. The time for the approach outlined in this article EM excludes the time needed for the initial geometry fit. Computations were performed with MATLAB R2023b in parallel on a workstation with a 2.70 GHz 6-core processor. There is no meaningful difference in memory usage between the different methods. RANSAC was limited to a maximum of a 100 point cloud subsets to evaluate. Meanwhile, the expected Mahalanobis distance approach used 1000 tangent lines and a maximum of 10 point cloud distribution updates. The number of tangent lines has relatively little effect on the computational time; however, the time scales linearly with the number of distribution updates.

## 5. Discussion

In this paper, we proposed a method to reconstruct geometry by transforming a point cloud into a fuzzy point cloud consisting of distributions of known form. We, however, note that in reality the distributions cannot be known exactly. For the laser scanning example, several factors such as vibration and wind will affect the uncertainty of each point but are unknown. Furthermore, as the scanners are made by commercial companies the hit-registration algorithm is not public, and thus, its uncertainty is similarly unknown. Additionally, while the effect of the incidence angle is taken into account, the cylinder example assumes a fully smooth surface. The roughness that exists in reality may be modeled statistically, for instance, as a Gaussian where the standard deviation and auto-correlation govern the height and planar distribution, respectively [17].

We note that the fuzzy geometry fitting approach such as described in this paper has an implicit dependence of the uncertainty on the geometry and is therefore an advancement over other methods found in literature [1,2,3,5]. It however has the inherent disadvantage that it requires a robust initial geometry estimate. If the initial estimate is highly erroneous the initial fuzzy point cloud estimate is likely to be erroneous as well and convergence of the iterative uncertainty updating procedure described in Section 3.3 is not guaranteed.

An aspect that is not considered in this paper is that a lack of points does not necessarily mean a lack of geometry but may instead be due to occlusion. The absence of evidence is thus not evidence of absence if the laser beam’s trajectory is obstructed. This can happen either due to the geometry itself (i.e., the back half of a cylinder) or due to other objects. We are unable to distinguish between areas devoid of points due to a lack of geometry and areas where points might have been were the area not obscured. The effect of occlusion on complex geometrical objects such as trees is significant and incorporating this into any analysis may greatly improve the results [18].

In this paper, the expected Mahalanobis distance E[M] is chosen over the expected Euclidean distance E[D]. Section 3.2 mentioned the general benefits that it is unitless, scale-invariant, and anisotropic. The scale dependence of E[D] induces bias that is easiest to explain when the distributions lie on the geometry. Generally speaking, E[D]∝|∑|; thus, the objective function would be biased to points with greater uncertainty, i.e., greater covariance. Similarly, bias due to isotropy is easiest to explain with the case of a line *L* where $\hat{\mu }\in L$. E[D] is then minimal when the fitted line $L_{\mathrm{fit}}$ is parallel to the principal axis $PC_{\sigma }$ of the covariance matrix. This bias decreases with the number of points when the coincidence of the points to the fitted line and the parallelity of the principal axes $PC_{\sum }\|L_{\mathrm{fit}}$ are mutually exclusive.

Bias is introduced for curved geometry, as the distance to a convex shape is always smaller from the inside than outside. In other words, this means that for a circle *C* with radius *r* as an example with $\hat{\mu }\in C$, $P_{\mathrm{out}}>P_{\mathrm{in}}$ leading to $r_{\mathrm{fit}}>r$ if the expected distance is taken to the curved shape directly. This further motivates our choice for approximating the shape by an envelope of tangent lines. An alternative approach is given in [5] which includes a term in the objective function that compensates for this bias. Such an approach was tested by the authors; however, it requires an accurate estimate of the geometry and was found to reduce robustness.

The interior-point algorithm used to minimize the expected Mahalanobis distance cylinder fitting objective requires a locally convex space to determine the update step, i.e., a positive-definite Hessian, which is not generally the case for our optimization problem. Instead, a positive-definite approximation of the Hessian is then used, meaning that the optimization problem the optimizer solves can deviate from the one specified. As such then, the optimizer sometimes finds a solution that may not be locally convex per the real Hessian. An alternative approach that does not approximate the Hessian may thus yield different results.

The approach described in this article provides an alternative to the least squares approach commonly used, and the average absolute error is lower for the geometry parameters of each of the three scanning situations. It is, however, important to note that the relative uncertainty in these results is often higher, and therefore, the performance difference is not uniformly statistically significant (*p* < 0.05). Additionally, the computational time of least squares without RANSAC was significantly smaller. It is important to note that for our approach the computational time scales roughly linearly with the number of points and the number of point cloud distribution updates. The requirement for such updates may, however, be relaxed when the number of points is higher or the noise level is lower, for instance, for the scanning case at 50 m. For simplicity, this was not taken into account in this study.

RANSAC in combination with least squares was implemented as a simpler approach to deal with noisy point cloud data; however, it does not consistently outperform least squares over the full point cloud and does not provide a benefit as an initial geometry fitting approach.

We further note that the cylinder vector has to be determined accurately for the cross-sectional circle fitting approaches of both least squares and our approach to be evaluated. As expected, the highest vector fitting error was found at 100 m due to the reduced quality and quantity of data, with the highest absolute error for least squares of 8.1% and for our approach of 4.2%, which is deemed acceptable by the authors.

The radius of cylinders scanned by laser data is commonly overestimated [14,19,20], likely because the aforementioned curvature-induced bias means points are on average located outside the surface. Least squares consistently overestimate the radius, and to a greater extent when the level of noise is greater (6.6% to 32.8%). The outlined approach is able to overcome this bias to a large extent with a statistically significant difference in error from −1.1% to 18.9%.

The case of two scanners at different ranges and different orientations was chosen in particular to test the proposed method. Ideally, combining a high- and low-quality data set would lead to a better result even if the average data quality deteriorates. The performance of the proposed method is, however, not uniformly better compared with using exclusively the higher quality data. The difference with respect to least squares (with and without RANSAC) is, however, statistically significant for all variables. It is further interesting to note that for least squares the error is roughly equal in the direction towards either scanner, while with our method it is clearly lower in the direction towards the closer (more certain) scanner.

Finally, it is of interest to see how the approach performs for other geometric shapes, and more work is needed to evaluate the approach more generally, such as for shapes of non-constant cross-sections or with asymmetrical curvature.

## 6. Conclusions

We presented a fuzzy point cloud data concept, which is a cloud of distributions instead of points, and a conceptual approach to using fuzzy point clouds in geometric shape fitting. The objective function consists of the average expected distance from the data points to the shape using the Mahalanobis distance instead of the Euclidean distance. Fuzzy point clouds model the uncertainty in the measurements and incorporate the possibility that the uncertainty can vary significantly in size and shape from point to point. This is the situation in laser scanning measurements in a complex environment such as forests. We demonstrated the approach with cylinder fitting to simulated laser scanner data, and the results show that the approach has a consistently lower average error than least squares fitting, with and without RANSAC. 

## Figures and Tables

**Table 1:** Relative cylinder fitting error ν and uncertainty σ. The cylinder (solid blue dot) is located at the origin.

		Center*_x_*		Center*_y_*		Vector*_x_*		Vector*_y_*		Radius	
		ν (%)	σ (%)	ν (%)	σ (%)	ν (%)	σ (%)	ν (%)	σ (%)	ν (%)	σ (%)
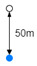	Scanner at (0, 50, 0)										
	ELS	1.41	5.82	24.2	17.2	−0.82	4.19	0.64	5.16	6.58	9.80
	RELS	1.82	7.10	18.1	17.6	−1.06	4.24	0.90	2.89	7.07	12.7
	EM	−0.92	12.8	9.90	27.4	−0.49	6.79	0.53	6.55	−1.07	16.2
	REM	−1.08	11.4	8.32	26.1	−0.19	6.59	0.55	5.27	−0.35	16.0
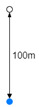	Scanner at (0, 100, 0)										
	ELS	12.6	57.1	52.0	101	−8.12	20.5	6.85	15.6	32.8	90.6
	RELS	11.4	55.7	59.8	107	−6.47	21.2	6.53	16.1	27.6	93.6
	EM	3.87	47.3	41.6	120	−1.76	20.0	1.99	15.6	14.5	103
	REM	1.15	51.3	49.7	133	−4.17	21.6	2.18	16.2	18.9	114
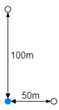	Scanners at (0, 100, 0) and (50, 0, 0)										
	ELS	9.70	12.0	9.36	6.82	−0.89	2.54	0.89	3.69	11.4	6.55
	RELS	7.25	12.3	7.88	7.50	−0.64	2.22	1.47	4.10	9.80	8.53
	EM	1.40	14.2	4.05	11.0	0.06	3.58	−0.19	6.99	4.28	10.2
	REM	2.05	16.4	4.36	11.2	0.05	4.35	0.20	6.16	3.80	11.3

**Table 2:** Probability values *p* according to the dependent pairwise Student’s *t*-test. The number of digits is such that *p* < 0.005, i.e., ten times lower than our limit for statistical significance, is shown as 0.00.

	Center*_x_*	Center*_y_*	Vector*_x_*	Vector*_y_*	Radius
Scanner at (0, 50, 0)					
*p* (ELS vs. EM)	0.01	0.00	0.41	0.76	0.00
*p* (RELS vs. REM)	0.00	0.00	0.04	0.34	0.00
Scanner at (0, 100, 0)					
*p* (ELS vs. EM)	0.01	0.31	0.00	0.00	0.00
*p* (RELS vs. REM)	0.01	0.22	0.26	0.00	0.03
Scanners at (0, 100, 0) and (50, 0, 0)					
*p* (ELS vs. EM)	0.00	0.00	0.00	0.01	0.00
*p* (RELS vs. REM)	0.00	0.00	0.02	0.00	0.00

**Table 3:** Computational time in seconds for the three scanning situations and tested methods.

	ELS	RELS	EM
Scanner at (0, 50, 0)	10.65 s	909.8 s	1993 s
Scanner at (0, 100, 0)	11.45 s	953.9 s	576.0 s
Scanners at (0, 100, 0) and (50, 0, 0)	10.50 s	527.2 s	2783 s

## Data Availability

The MATLAB R2023b code is available online at https://github.com/InverseTampere/Cylinder_Fitting, accessed on 29 December 2024.

## Appendix A

Due to the constant cross-sectional shape, the problem can be simplified by projecting the three-dimensional (3D) Gaussians onto the cross-sectional plane, where the z-axis is parallel to the cylinder axis, $Q⊥\vec{v}\equiv \vec{z}$, resulting in a two-dimensional (2D) Gaussian.

The exponential term of the probability density function for an arbitrary Gaussian $N(\hat{\mu },\sum )$ can be rewritten to an ellipsoidal parametrization such that it can be directly expressed as a function of the Cartesian coordinate system, in this case, x,y,z. The ellipsoidal representation is shown in Equations (A1)–(A3) using the precision matrix $P=\sum^{-1}$. The indices denote those dimensions’ entry; thus, $P_{xx}$, $P_{yy}$, $P_{zz}$, $P_{xy}$, $P_{xz}$, and $P_{yz}$ are elements of the precision matrix *P*.

(A1) $$\begin{array}{c} \begin{array}{cc} f(\hat{x}) & =\frac{e^{-\frac{1}{2}{\left(\hat{x}-\hat{\mu }\right)}^{T}\sum^{-1}\left(\hat{x}-\hat{\mu }\right)}}{\sqrt{8\pi^{3}\left|\sum \right|}}\\ \; & =Ke^{-\frac{1}{2}{\left(\hat{x}-\hat{\mu }\right)}^{T}\sum^{-1}\left(\hat{x}-\hat{\mu }\right)}\\ & =Ke^{-\frac{1}{2}\left(P_{xx}x^{2}+P_{yy}y^{2}+P_{zz}z^{2}\right)+Ax+By+Cz-P_{xy}xy-P_{xz}xz-P_{yz}yz+D}\\ K & =\frac{1}{\sqrt{8\pi^{3}\left|\sum \right|}}, \end{array} \end{array}$$

The constants A,B,C, and *D* are given by Equations (A2) and (A3):

(A2) $$\left[\begin{array}{c} A\\ B\\ C \end{array}\right]=P\hat{\mu }$$

(A3) $$\begin{array}{c} D=-\frac{1}{2}\left(P_{xx}{\hat{\mu }}_{x}^{2}+P_{yy}{\hat{\mu }}_{y}^{2}+P_{zz}{\hat{\mu }}_{z}^{2}\right)-\left(P_{xy}{\hat{\mu }}_{x}{\hat{\mu }}_{y}+P_{xz}{\hat{\mu }}_{x}{\hat{\mu }}_{z}+P_{yz}{\hat{\mu }}_{y}{\hat{\mu }}_{z}\right) \end{array}$$

The marginal distribution f(x,y) on the plane is the integral of the probability density with respect to *z*, which due to the ellipsoidal parametrization can be derived easily as shown in Equation (A4). The expected value and precision matrix of the projected 2D Gaussian are denoted by $\mu_{Q}$ and *Q*, respectively.

(A4) $$\begin{array}{c} f\left(x,y\right)=\int_{R}f\left(\hat{x}\right)dz=K\sqrt{\frac{2\pi }{P_{zz}}}e^{-\frac{1}{2}{\left(\hat{x}-{\hat{\mu }}_{Q}\right)}^{T}Q\left(\hat{x}-{\hat{\mu }}_{Q}\right)} \end{array}$$

The dimensionality reduction is also evident by the parametrization of a 2D ellipse in the exponential with constants α through ω.

(A5) $$\begin{array}{c} \begin{array}{cc} \; & -\frac{1}{2}{\left(\hat{x}-{\hat{\mu }}_{Q}\right)}^{T}Q\left(\hat{x}-{\hat{\mu }}_{Q}\right)\\ \; & =\frac{1}{2}\left(\frac{P_{xz}^{2}}{P_{zz}}-P_{xx}\right)x^{2}\\ \; & +\frac{1}{2}\left(\frac{P_{yz}^{2}}{P_{zz}}-P_{yy}\right)y^{2}+\left(\frac{P_{xz}P_{yz}}{P_{zz}}-P_{xy}\right)xy\\ \; & +\left(A-C\frac{P_{xz}}{P_{zz}}\right)x+\left(B-C\frac{P_{yz}}{P_{zz}}\right)y+\frac{C^{2}}{2P_{zz}}+D\\ \; & =\alpha x^{2}+\beta y^{2}+\gamma xy+\delta x+\epsilon y+\omega \end{array} \end{array}$$

From this, it follows that the precision matrix equals $Q=-\left[\begin{array}{c} 2\alpha \;\gamma \\ \gamma \;2\beta \end{array}\right]$.

Its inverse is the 2D covariance matrix

(A6) $$\sum_{Q}=Q^{-1}=\frac{1}{\gamma^{2}-4\alpha \beta }\left[\begin{array}{c} 2\beta \;-\gamma \\ -\gamma \;2\alpha \end{array}\right]=\psi \left[\begin{array}{c} 2\beta \;-\gamma \\ -\gamma \;2\alpha \end{array}\right].$$

Its two eigenvectors $v_{1,2}$ form the projected Gaussian axes, and its eigenvalues $\lambda_{1,2}$ are the variances along said axes given by (A7) and (A8), respectively.

(A7) $$\begin{array}{c} {\vec{v}}_{1,2}=\frac{1}{\sqrt{\psi^{2}\gamma^{2}+{\left(2\psi \beta -\lambda_{1,2}\right)}^{2}}}\left[\begin{array}{c} \psi \gamma \\ 2\psi \beta -\lambda_{1,2} \end{array}\right] \end{array}$$

(A8)
$$\sigma_{1,2}^{2}=\lambda_{1,2}=\psi \left(\alpha +\beta \pm \sqrt{\alpha^{2}+\beta^{2}+\gamma^{2}-2\alpha \beta }\right)$$
